# Cocoon vaccination for influenza in patients with a solid tumor: a retrospective study

**DOI:** 10.1007/s00520-020-05883-2

**Published:** 2020-11-12

**Authors:** M. J. Rensink, H. W. M. van Laarhoven, F. Holleman

**Affiliations:** 1 Department of Internal Medicine, Amsterdam University Medical Centers (UMC), University of Amsterdam, Meibergdreef 9, 1105 AZ Amsterdam, The Netherlands; 2 Department of Medical Oncology, Cancer Center Amsterdam, Amsterdam University Medical Centers (UMC), University of Amsterdam, Meibergdreef 9, 1105 AZ Amsterdam, The Netherlands

**Keywords:** Vaccination, Cocoon vaccination, Influenza, Household contacts, Oncological patients, Retrospective study

## Abstract

**Purpose:**

Oncological patients are susceptible to various severe viral infections, including influenza. Vaccinating oncological patients and their household contacts (“cocoon vaccination”) may protect these patients from contracting influenza. To understand the potential of cocoon vaccination in oncological patients, this study assesses the influenza vaccination status of oncological patients and their household contacts and their considerations regarding the vaccination.

**Methods:**

In this retrospective study, oncological patients with a solid tumor were asked to fill in a questionnaire about their own and their household contacts’ influenza vaccination status in the influenza season of 2018–2019.

**Results:**

Ninety-eight patients were included (response rate 88%). The influenza vaccination rates of oncological patients and their first household contacts were 43.9% and 44.9%, respectively. The majority of vaccinated patients and vaccinated first household contacts had been advised by their general practitioner to get the vaccination. A minority of the first household contacts reported getting vaccinated specifically because of the patient’s vulnerability. Unvaccinated patients and unvaccinated household contacts mainly believed the vaccination was unnecessary or were afraid of side effects. None of the included patients had been hospitalized with influenza.

**Conclusion:**

The oncological patients’ and first household contacts’ vaccination rates in this study were lower than the vaccination rates of the general Dutch population of over 60 years old, possibly due to a lack of knowledge and misconceptions about the vaccination. Further research is required to establish whether cocoon vaccination can contribute to protecting oncological patients from contracting an influenza infection.

**Supplementary Information:**

The online version contains supplementary material available at 10.1007/s00520-020-05883-2.

## Introduction

Viral infections have a serious impact on people’s health worldwide, as is evidenced by the current COVID-19 (coronavirus disease 2019) pandemic that, at the time of writing, has resulted in over a million deaths worldwide and for which a vaccine is urgently awaited [1]. Vaccines are, however, available for influenza, and yet, according to the World Health Organization (WHO), three to five million patients globally suffer from a severe influenza infection every year, resulting in approximately 290,000 to 650,000 deaths [2]. A recent study suggests that this may even be an underestimation of the actual number of deaths caused by influenza infections each year [3]. In the Netherlands, people of 60 years and older and patients with comorbidities who are vulnerable to serious influenza infections are advised to get vaccinated by their general practitioner (GP) [4]. However, for various reasons, not all patients are willing to receive the vaccination. The vaccination rate of people older than 65 years in the influenza season of 2018–2019 in the Netherlands was 62.7%, which is the third highest rate of the European Union, following the UK (72.0%) and Ireland (68.5%) [4, 5]. Yet the WHO goal of a 75% vaccination rate in patients older than 65 years has not been reached [2].

Elderly and immunocompromised patients, such as oncological patients receiving chemotherapy, are more susceptible to serious influenza infections, which means that they can possibly benefit more from being vaccinated [6]. Their immune response however is less adequate which makes the vaccination less effective [7–11]. This means that these individuals may need additional protection, for example, by vaccinating healthcare workers, because they are in close contact with patients highly susceptible to influenza [12–17]. Unfortunately, the vaccination rate of healthcare workers is generally low in the Netherlands. In a 2012 study, the vaccination rate of hospital personnel in the Netherlands was 2–33%, with a median of 12% [18]. The average vaccination rate of healthcare workers in Europe in 2016–2017 was 30.2% [19]. This percentage is insufficient to reduce the spread of influenza substantially. Moreover, this percentage has not really increased over the years [20, 21]. This is in sharp contrast with the vaccination rates of healthcare workers in the USA. After implementing a mandatory vaccination program, vaccination rates of hospital personnel increased from 56 (2006–2007) to 94% (2013–2014). This was associated with a significant reduction of nosocomial infections in immunocompromised cancer patients [16].

The closest contacts of vulnerable patients are often the people they live with. Unsurprisingly, household contacts of patients can also transmit the virus to the patients [22]. The recent COVID-19 pandemic has shown that close contacts have an important role in spreading viral infections. In Australia in May 2020, the number of influenza infections had decreased to 1% of the infections present in May 2019 [23]. The coronavirus measures, such as physical distancing and advice about extra hygiene, seem to have helped reduce the transmission of viral infections. In the case of influenza, this implies that vaccinating household contacts and thereby decreasing the chance of household contacts getting an influenza infection can possibly protect vulnerable patients [24]. This strategy is called cocoon vaccination. Data from several studies suggest that cocoon vaccination can be effective in decreasing the chance of infants contracting *Bordetella pertussis* [25, 26]. Furthermore, a Canadian study showed that vaccinating children can protect unvaccinated adults from contracting influenza infections [27]. To our knowledge, no previous studies have researched whether cocoon vaccination against influenza protects oncological patients from contracting a serious influenza infection.

In this study, we assessed the vaccination status of oncological patients and their household contacts to understand the potential of cocoon vaccination in this population. Additionally, we investigated the considerations of patients and their household contacts regarding the decision to vaccinate against influenza or not.

## Methods

### Design and research population

This retrospective study was conducted at the Department of Internal Medicine at the Academic Medical Center (AMC), one of the Amsterdam University Medical Centers hospitals. After a data privacy impact assessment, the Data Privacy Office approved the study. From April to July 2019, adult oncological patients were invited by two assessors to take part in the study. Over this period, the assessors randomly chose different moments of alternating days to visit the oncology outpatient clinic and the chemotherapy treatment center. All the patients present at the time were asked to participate in the study. Patients who were approached received an information letter about the study and a questionnaire. They were also asked to give informed consent to the collection of their relevant medical history for this study.

**Fig. 1:**
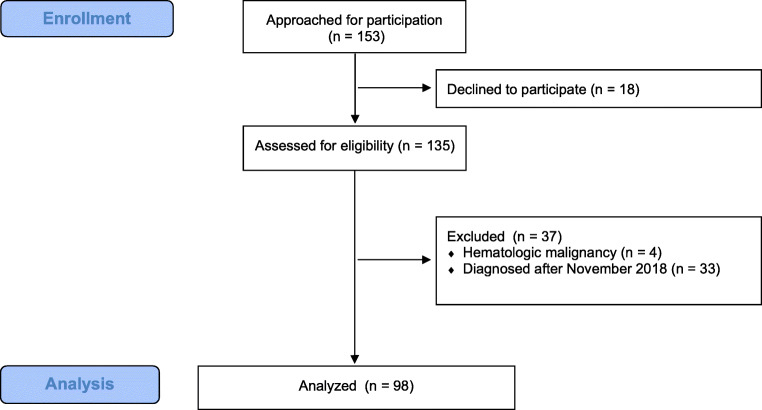
Consort flow diagram

This study researched oncological patients with a solid tumor of 18 years and older. We excluded patients with a hematologic malignancy as hematological illnesses have a specific relationship with the immune system and the influenza vaccination may have a different effect on this group of patients. This subject was beyond the scope of this study and needs to be researched in future studies. We also excluded patients who did not speak the Dutch or English language sufficiently and patients who were diagnosed with cancer after November 2018 (Fig. 1). The reason for this is that the 2018 influenza vaccination in the Netherlands was administered by GPs up until November of that year [28]. Therefore, patients diagnosed after November would not have taken their illness into consideration when making a decision about getting vaccinated. In the Netherlands, vulnerable patients and patients of 60 years and older receive a letter from their GP every year with the recommendation to get vaccinated. The GP has the role of advocate for the influenza vaccine and is also responsible for giving advice and the administration of the vaccination. There is no protocol for oncologists regarding influenza vaccination advice for patients. Healthy household contacts are not routinely advised to get vaccinated.

Patients were also asked to fill out information about their household contacts. A household contact was defined as a person they lived with. We named the patients’ partner the “first household contact.” If a patient did not have a partner, the first household contact was the first person the patient mentioned. The following household contacts that the patient mentioned were named respectively “second household contact” to “fifth household contact.”

A Dutch questionnaire was specifically written and used for this study (translated to English in the Supplementary Material). The majority of the patients filled out the questionnaire themselves. Some patients preferred to answer the questions orally while the researcher transcribed their answer. The questions were asked and answered orally in English if patients did not understand the Dutch language sufficiently.

### Questionnaire variables

The patient questionnaires were used to collect information on the patients’ general characteristics, influenza vaccination status, reasons for (not) being vaccinated, and the number of household contacts.

If patients had household contacts, they were asked to fill in additional questionnaires, one for each of their household contacts. The additional questionnaires collected information on the household contact’s general characteristics, influenza vaccination status, reasons for (not) being vaccinated, and the role of the patient’s illness in this decision.

### Medical file information

One assessor (M.R.) consulted the Electronic Patient Records. He examined whether the patients had been tested positive for influenza in the AMC in the 2018–2019 influenza season (December until March) and assessed the patient’s type of oncological disease, the date of diagnosis, and the treatment.

### Statistical analysis

All questionnaires were anonymized after completion. We statistically analyzed the data with the use of SPSS version 26.0.0.0. Logistic regression was used to assess the risk factors of non-vaccination for patients and their household contacts. Significance was determined by a *p* value lower than 0.05. The factors “age” and “sex” were studied in the patient and household contact group. We did not include the variable “living situation” because only a small number of patients did not live independently. The variable “vaccinated in the past 5 years” was not added to the analysis because this was too closely related to the vaccination status. We did not include the variable “invited by the GP for the influenza vaccination” since the recommendation had taken place approximately 6 months before participants answered the questionnaire. It is therefore possible that unvaccinated patients were less likely to remember receiving an automated letter with vaccination advice from their GP than vaccinated patients.

## Results

**Fig. 2:**
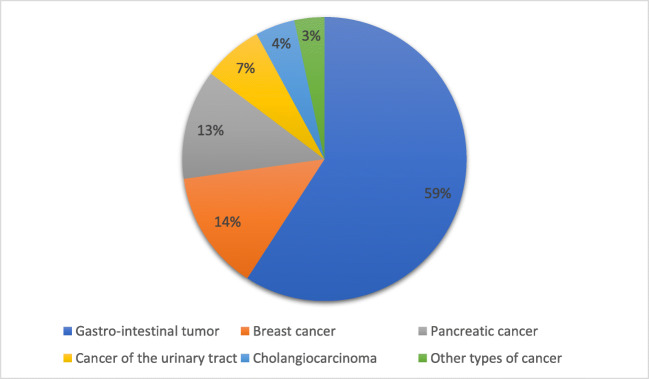
Patients’ diagnosis

In total 135 patients filled in the questionnaire (response rate 88%, Fig. 1). We excluded four patients with a hematologic malignancy. In addition, 33 patients that were diagnosed with cancer after November 2018 were excluded. Ninety-eight patients were analyzed using the data obtained from their questionnaires. Of the 98 included patients, 88 patients also gave informed consent to consult their Electronic Patient Records. We were therefore able to obtain the following additional information about the 88 patients that gave us access to their medical records: 62.5% were undergoing palliative treatment and 37.5% were undergoing curative treatment. A gastro-intestinal tumor was diagnosed in 59.1% of the patients. Other patients were diagnosed with breast cancer (13.6%), pancreatic cancer (12.5%), cancer of the urinary tract (6.8%), cholangiocarcinoma (4.5%), and other types of cancer (3.4%, Fig. 2).

### Population characteristics

The vaccinated patients were older than the unvaccinated patients (mean 67.0 vs. 62.0 years old, Table 1). Similarly, the vaccinated first household contacts were older than the unvaccinated first household contacts (> 60 years old: 83.9% vs. 39.5%, Table 2). Most of the patients lived independently (90.8%). Approximately one-third of the patients lived alone, and about half of the patients only had one household contact. If patients lived with one or more household contacts, the first household contact was almost always the partner (94.0%). Of the 33 other household contacts (second to fifth household contacts), 32 were not vaccinated (97%). Of these 33 household contacts, 30 (91%) were the patients’ children (90% aged 0–20 years). The 32 unvaccinated second to fifth household contacts had either not been invited by their GP to be vaccinated (73%) or considered vaccination unnecessary (27%).

**Table 1:** Vaccinated versus unvaccinated patients

	Vaccinated patients (*n* = 43)	Unvaccinated patients (*n* = 55)	OR (95% CI)	*p* value
	% (*n*)	% (*n*)		
Sex				
Female	48.8% (21)	45.5% (25)	1.046 (0.459–2.383)	0.914
Male	51.2% (22)	54.5% (30)	1	
Age (mean in years)	67.0 range 38–81	62.0 range 39–91	1.042 (1.003–1.083) per year of increased age	0.035
Living situation				
Independent	86.0% (37)	94.5% (52)		
With informal care	7.0% (3)	3.6% (2)		
With home care	4.7% (2)	1.8% (1)		
In a nursing home	2.3% (1)	0		
Household contacts				
None	30.2% (13)	27.3% (15)		
1	53.5% (23)	45.5% (25)		
2 or more	14.0% (6)	27.3% (15)		
I live in a nursing home	2.3% (1)	0		
Invited by the GP for the influenza vaccination	95.3% (41)	54.5% (30)		
Vaccination advised by (multiple answers possible)				
The GP	28			
The oncologist	7			
The media	1			
Own initiative	11			
Other	1			
Reason(s) for being vaccinated (multiple answers possible)				
Because of my own health	33			
Because I was advised to	17			
To protect others	1			
I don’t know	1			
Other	1			
Reason(s) for not being vaccinated (multiple answers possible)				
I did not receive an invitation from my GP		10		
I do not find it necessary		26		
I am principally against vaccinations		1		
I am afraid of side effects		17		
I forgot about the vaccination		6		
I do not get the flu		3		
Other		5		
Vaccinated in the past 5 years	42 patients (1 missing)			
Yes, every year	58.1% (25)	5.5% (3)		
Yes, 2–4 times	11.6% (5)	3.6% (2)		
Yes, once	14.0% (6)	14.5% (8)		
No	14.0% (6)	76.4% (42)		
Do you take special measures to prevent catching the flu during the flu season, such as washing your hands more often or having less physical contact when greeting someone?				
Yes, always	20.9% (9)	27.3% (15)		
Yes, when I have guests	11.6% (5)	3.6% (2)		
Yes, but only in the hospital	7.0% (3)	3.6% (2)		
Yes, when I have guests and in the hospital	4.7% (2)	1.8% (1)		
Yes, other	0	5.5% (3)		
No	55.8% (24)	58.2% (32)

**Table 2:** Vaccinated versus unvaccinated first household contacts

	Vaccinated first household contacts (*n* = 31)	Unvaccinated first household contacts (*n* = 38)	OR (95% CI)	*p* value
	% (*n*)	% (*n*)		
Sex				
Female	48.4% (15)	65.8% (25)	0.623 (0.214–1.809)	0.384
Male	51.6% (16)	34.2% (13)	1	
Age			1.888 (1.231–2.895) per 10 years of increased age	0.004
> 60 years old	83.9% (26)	39.5% (15)		
Relationship with the patient				
Partner	96.8% (30)	92.1% (35)		
First degree relation (child, parent)	3.2% (1)	7.9% (3)		
Advised by a GP for the influenza vaccination				
Yes	96.8% (30)	36.8% (14)		
Reason(s) for being vaccinated (multiple answers possible)				
Because of the patient’s health	10			
Because of the household contact’s own health	22			
For work	3			
Other	1			
Reason(s) for not being vaccinated (multiple answers possible)				
The household contact did not receive an invitation from the GP		10		
The household contact thought it was unnecessary		19		
The household contact is principally against vaccinations		1		
The household contact is afraid of side effects		6		
The household contact forgot about the vaccination		3		
Other		2		
If household contacts were vaccinated for their own health, this had been advised by	22 patients			
The GP	86.4% (19)			
Own initiative	9.1% (2)			
Other	4.5% (1)			
If household contacts were vaccinated for the patient’s health, this had been advised by	10 patients			
The GP	40% (4)			
The oncologist	20% (2)			
Own initiative	30% (3)			
All of the above	10% (1)			
Vaccinated in the past 5 years		36 patients (2 missing)		
Yes, every year	74.2% (23)	5.3% (2)		
Yes, 3 times	-	2.6% (1)		
Yes, once	12.9% (4)	7.9% (3)		
No	12.9% (4)	78.9% (30)		

### Influenza vaccination

**Fig. 3:**
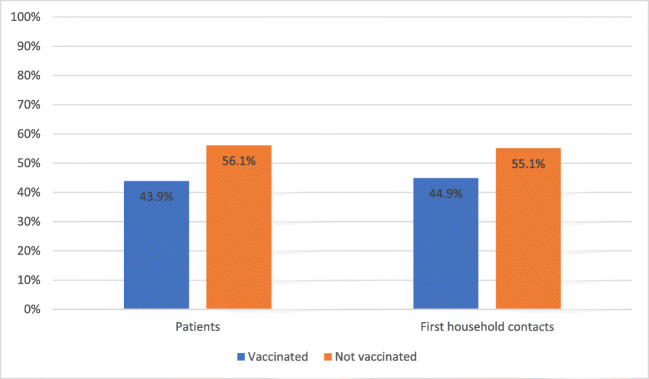
Vaccination coverage in influenza season 2018–2019

The influenza vaccination rate of the patients in this study was 43.9%, and the vaccination rate of the first household contacts was 44.9% (Fig. 3). Of the 43 vaccinated patients, 29 had at least one household contact (67.4%), and 26 of these 29 first household contacts had also been vaccinated (89.7%). Of the vaccinated patients and vaccinated first household contacts, the majority was 60 years or older (respectively, 76.7% and 83.9%), which means that they would have been advised to get vaccinated solely because of their age. Of the unvaccinated patients and unvaccinated first household contacts, 61.8% and 39.5% were 60 years or older. In the category of patients of 60 years and older, 49.3% was vaccinated.

**Fig. 4:**
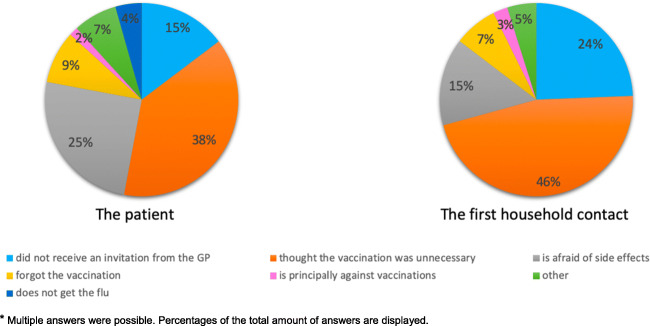
Patients’ and first household contacts’ reasons for not getting vaccinated

Almost all vaccinated patients and vaccinated first household contacts indicated that they had been advised to get the vaccination by their GP (respectively, 95.3% and 96.8%). The majority of the vaccinated patients and first household contacts had been vaccinated every year in the previous 5 years (respectively, 58.1% and 74.2%). Among the unvaccinated patients and unvaccinated first household contacts, there was a lower percentage of people that reported having received a recommendation from their GP to get vaccinated (respectively, 54.5% and 36.8%). The unvaccinated patients and unvaccinated household contacts were more likely not to have had any vaccination in the previous 5 years (respectively, 76.4% and 78.9%). Patients mainly chose to receive the vaccination for their own health (76.7%) and because they had been advised to do so (39.5%). If patients refused the vaccination, this was often because they thought the vaccination was unnecessary (47.3%) or because they were afraid of side effects (30.9%, Fig. 4). Regarding the reasons for vaccination, the vaccinated first household contacts most often mentioned that they had chosen to get vaccinated for their own health (71.0%) and to a lesser extent because of the health of the patient (32.3%). Unvaccinated first household contacts often thought vaccination was unnecessary (50.0%, Fig. 4).

### Hospitalization with an influenza infection

None of the included patients had been hospitalized with a proven influenza infection in the influenza season of 2018–2019.

### Logistic regression

Risk factors for non-vaccination were assessed for patients and their household contacts. The older the patient or first household contact, the higher the likelihood of being vaccinated. The odds of the patient being vaccinated increased by 1.042 per year of increased age (95% CI 1.003–1.083, Table 1). The household contact’s likelihood of being vaccinated increased by 1.888 per 10 years of increased age (95% CI 1.231–2.895, Table 2). Due to privacy reasons, the household contact’s age was analyzed per 10 years instead of 1 year. In both the patients’ and household contacts’ groups, sex was not a significant risk factor for non-vaccination.

## Discussion

This is the first study in a European country on the vaccination status of both adult oncological patients and their household contacts and their reasons for (not) getting vaccinated.

The vaccination rates of oncological patients in our study are lower than in a previous Dutch study in 2013, which showed an influenza vaccination rate of 59% in oncological patients [29]. This is alarming, also in view of the current COVID-19 pandemic and the hopes that a vaccine may counteract the devastating effects of the coronavirus. Oncological patients are more susceptible to contracting a serious influenza infection due to their illness, a reduced immune system during and post (chemotherapy) treatment, and because of their regular hospital visits [10, 16, 30]. Despite the large number of influenza deaths and the oncological patients’ higher risk of contracting a serious influenza infection, vaccination is still frequently declined by these patients and their household contacts.

A 2019 study in the USA investigated the influenza vaccination status of oncological patients and their caregivers [31]. The vaccination rate among patients was 72% and among caregivers 71%. The oncological patients in the USA study considered it important for their health that their caregivers got vaccinated, while the caregivers’ decision to be vaccinated did not appear to be affected by the patients’ disease. Likewise, in our study, the majority of first household contacts did not seem to take the vulnerability of the oncological patient into account when deciding to get vaccinated. Only 32.3% of the vaccinated first household contacts got the vaccination to protect the patients’ health.

Unvaccinated patients and unvaccinated first household contacts mainly indicated that they thought they did not need the vaccination or were afraid of side effects. They often noted that they were afraid the vaccination would make them ill. Likewise, a 2020 study displayed that 20% of the at-risk patients had concerns about the safety of the influenza vaccination [32]. A large Toronto study showed, however, that only 5.8% of the 670 participants had influenza-like symptoms after getting vaccinated [33]. There were no severe side effects reported. Accordingly, if patients are informed that the risk of getting symptoms caused by the vaccination is very low, it may increase the likelihood of them choosing to get vaccinated. Healthcare providers should pay attention to this topic when informing patients and their household contacts about the vaccination. Not all patients will be influenced by receiving first-hand information [34], but earlier studies have shown that giving patients high-quality information about the vaccination raises the vaccination rates [35, 36].

The majority of the patients (65.1%) and first household contacts (77.4%) got vaccinated at the recommendation of their GP, while only seven patients and three household contacts were vaccinated at the recommendation of the patients’ oncologist. Oncologists, often the primary doctors of oncological patients, can possibly play a larger role in advising patients and household contacts to get vaccinated. An earlier study has shown that vaccination advice from a rheumatologist was more effective than the advice from the GP in convincing patients to get vaccinated for influenza [37]. An oncologist’s recommendation for the influenza vaccination may therefore possibly increase the vaccination rate of oncological patients. In addition, a 2019 French study investigated the considerations of GPs regarding the influenza vaccination for oncological patients. The study showed that GPs wanted to have more contact with the patient’s oncologist in order to give the best influenza vaccination advice to the patient [38].

The influenza vaccination rate of the oncological patients of 60 years and older was 49.3%, which is lower than the vaccination rate of the general Dutch population of 60 years and older (53.8% in 2018) [30]. Everyone in the Netherlands of 60 years and older receives an annual vaccination recommendation from their GP. The results of our study suggest that the additional vulnerability of oncological patients of 60 years and older does not heighten the vaccination rate of these patients. There is a potential for increasing the vaccination rate among oncological patients if oncologists advocate and give advice about the influenza vaccine as well as GPs.

The logistic regression that was conducted in our study presented being of a younger age as a significant risk factor for non-vaccination in patients and household contacts. This is unsurprising considering that older people often have more comorbidities and are more vulnerable than younger people. More focus should possibly be put on advising younger oncological patients to get vaccinated.

Interestingly, in our study sample, none of the 98 included patients had tested positive for influenza in the 2018–2019 influenza season, even though more than half of the patients and their household contacts had not been vaccinated. We could therefore neither confirm nor reject our hypothesis that vaccinating the household contacts of oncological patients can protect oncological patients from a serious influenza infection. The question is whether the vaccination of household contacts could be effective for preventing hospitalization of patients or alternatively whether the absolute risk of contracting influenza is too low to warrant large-scale vaccination. Earlier literature shows that oncological patients’ hospitalization rates and death rates attributed to influenza infections (0.44% and 0.04%) are generally four times higher than those reported in the general population [10]. Retrospectively, the influenza season of 2018–2019 was relatively mild in the Netherlands as the number of infections was 400,000 (approximately one in 43 people) [4]. This was lower than in the previous seasons (900,000 in 2017–2018, 500,000 in 2016–2017) [39, 40]. Further research on a larger scale has to be carried out to gather statistics on the number of oncological patients that contract a severe influenza infection and whether these patients can profit from vaccinated household contacts. Importantly, the recent Australian reports during the COVID-19 pandemic show that physical distancing and extra hygienic measures can possibly make a great difference in limiting the spread of influenza [23]. Further research is required to evaluate how these measures can contribute to decreasing influenza infection rates in oncological patients when the COVID-19 measures are lifted.

There were some limitations to this study. Firstly, solely Dutch and English-speaking patients were included, and a Dutch questionnaire was used. Two assessors worked on this research, and they transcribed the patients’ answers if they were done orally. This could have possibly led to some inter-observer discrepancies. Uncertainties were however discussed, and a standardized questionnaire was used. Secondly, it is possible that patients were not fully aware of their household contacts’ considerations regarding vaccination. The patients were asked to fill in their household contacts’ vaccination status and the reasons for getting vaccinated or not. The first household contacts were often present; however, in some cases, patients were alone. In this case, patients were able to leave the question open. Furthermore, 95.3% of the vaccinated patients remembered being advised to get vaccinated by their GP, while only 54.5% of the unvaccinated patients claimed to have received this recommendation. It is possible that unvaccinated patients may have forgotten that they received a recommendation from their GP as vulnerable patients receive an automated letter about the flu vaccination. Thirdly, our vaccination rates could be an underestimation of the general vaccination rate of all oncological patients. We focused solely on patients in the outpatient clinic and at the outpatient chemotherapy treatment center. Patients that receive chemotherapy clinically may be more vulnerable and may therefore be more likely to get vaccinated. Furthermore, more vulnerable patients may not have been willing or able to participate in this study. Finally, we may not have been able to include oncological patients that had declined or were not eligible for chemotherapy due to a poor prognosis as these patients visit the outpatient clinic or the treatment center less frequently. However, the influenza vaccination would not be substantially beneficial for these patients because of their poor prognosis. Their vaccination status was therefore not the main focus of this study.

More research has to be done to determine the exact burden of influenza in the oncological patient population and whether cocoon vaccination can contribute to protecting oncological patients from contracting an influenza infection. There is a possibility that the vaccination rate of patients and their household contacts could be raised by the oncologist bringing up the topic of vaccination and giving advice on it. GPs and oncologists should address patients’ and household contacts’ misconceptions about side effects.

## Supplementary information

ESM 1 (DOCX 22 kb).

## Data Availability

The data that support the findings of this study are available from the corresponding author, M.R., upon reasonable request.

## References

[1] Johns Hopkins Center for Systems Science and Engineering (2020) Coronavirus resource center: COVID-19 dashboard by the Center for Systems Science and Engineering (CSSE) at Johns Hopkins University (JHU). https://coronavirus.jhu.edu/map.html. Accessed 14 Oct 2020

[2] World Health Organisation (2018) Influenza (Seasonal) Fact sheet. https://www.who.int/news-room/fact-sheets/detail/influenza-(seasonal). Accessed 15 Nov 2019

[3] Iuliano AD, Roguski KM, Chang HH, Muscatello DJ, Palekar R, Tempia S, Cohen C, Gran JM, Schanzer D, Cowling BJ, Wu P, Kyncl J, Ang LW, Park M, Redlberger-Fritz M, Yu H, Espenhain L, Krishnan A, Emukule G, van Asten L, Pereira da Silva S, Aungkulanon S, Buchholz U, Widdowson MA, Bresee JS, Azziz-Baumgartner E, Cheng PY, Dawood F, Foppa I, Olsen S, Haber M, Jeffers C, MacIntyre CR, Newall AT, Wood JG, Kundi M, Popow-Kraupp T, Ahmed M, Rahman M, Marinho F, Sotomayor Proschle CV, Vergara Mallegas N, Luzhao F, Sa L, Barbosa-Ramírez J, Sanchez DM, Gomez LA, Vargas XB, Acosta Herrera B, Llanés MJ, Fischer TK, Krause TG, Mølbak K, Nielsen J, Trebbien R, Bruno A, Ojeda J, Ramos H, an der Heiden M, del Carmen Castillo Signor L, Serrano CE, Bhardwaj R, Chadha M, Narayan V, Kosen S, Bromberg M, Glatman-Freedman A, Kaufman Z, Arima Y, Oishi K, Chaves S, Nyawanda B, al-Jarallah RA, Kuri-Morales PA, Matus CR, Corona MEJ, Burmaa A, Darmaa O, Obtel M, Cherkaoui I, van den Wijngaard CC, van der Hoek W, Baker M, Bandaranayake D, Bissielo A, Huang S, Lopez L, Newbern C, Flem E, Grøneng GM, Hauge S, de Cosío FG, de Moltó Y, Castillo LM, Cabello MA, von Horoch M, Medina Osis J, Machado A, Nunes B, Rodrigues AP, Rodrigues E, Calomfirescu C, Lupulescu E, Popescu R, Popovici O, Bogdanovic D, Kostic M, Lazarevic K, Milosevic Z, Tiodorovic B, Chen M, Cutter J, Lee V, Lin R, Ma S, Cohen AL, Treurnicht F, Kim WJ, Delgado-Sanz C, de mateo Ontañón S, Larrauri A, León IL, Vallejo F, Born R, Junker C, Koch D, Chuang JH, Huang WT, Kuo HW, Tsai YC, Bundhamcharoen K, Chittaganpitch M, Green HK, Pebody R, Goñi N, Chiparelli H, Brammer L, Mustaquim D (2018). Estimates of global seasonal influenza-associated respiratory mortality: a modelling study. Lancet.

[4] Schurink-van ’t Klooster T, van Gageldonk-Lafeber A, Wallinga J, Meijer A, van Boven M, Sanders E, de Vos Klootwijk L et al (2019) Influenza vaccination in the Netherlands: Background information for the Health Council of the Netherlands. Retrieved from https://www.rivm.nl/bibliotheek/rapporten/2019-0002.pdf. 10.21945/rivm-2019-0002

[5] Organisation of Economic Co-operation and Development (OECD) (2016) OECD Statistics - Health Care Utilisation: immunisation. https://stats.oecd.org/index.aspx?r=37554. Accessed 10 Nov 2020

[6] Gruneir A, Kwong JC, Campitelli MA, Newman A, Anderson GM, Rochon PA, Mor V (2014). Influenza and seasonal patterns of hospital use by older adults in long-term care and community settings in Ontario, Canada. Am J Public Health.

[7] Kunisaki KM, Janoff EN (2009). Influenza in immunosuppressed populations: a review of infection frequency, morbidity, mortality, and vaccine responses. Lancet Infect Dis.

[8] Zheng B, Zhang Y, He H, Marinova E, Switzer K, Wansley D, Mbawuike I, Han S (2007). Rectification of age-associated deficiency in cytotoxic T cell response to influenza A virus by immunization with immune complexes. J Immunol.

[9] Targonski PV, Jacobson RM, Poland GA (2007). Immunosenescence: role and measurement in influenza vaccine response among the elderly. Vaccine.

[10] Cooksley CD, Avritscher EBC, Bekele BN, Rolston KV, Geraci JM, Elting LS (2005). Epidemiology and outcomes of serious influenza-related infections in the cancer population. Cancer.

[11] Zbinden D, Manuel O (2014). Influenza vaccination in immunocompromised patients: efficacy and safety. Immunotherapy.

[12] Burls A, Jordan R, Barton P (2006). Vaccinating healthcare workers against influenza to protect the vulnerable-Is it a good use of healthcare resources?. A systematic review of the evidence and an economic evaluation. Vaccine.

[13] Jenkin DC, Mahgoub H, Morales KF, Lambach P, Nguyen-van-Tam JS (2019). A rapid evidence appraisal of influenza vaccination in health workers: an important policy in an area of imperfect evidence. Vaccine X.

[14] Blanco N, Eisenberg MC, Stillwell T, Foxman B (2016). What transmission precautions best control influenza spread in a hospital?. Am J Epidemiol.

[15] Weinstein RA, Bridges CB, Kuehnert MJ, Hall CB (2003). Transmission of influenza: implications for control in health care settings. Clin Infect Dis.

[16] Frenzel E, Chemaly RF, Ariza-Heredia E, Jiang Y, Shah DP, Thomas G, Graviss L, Raad I (2016). Association of increased influenza vaccination in health care workers with a reduction in nosocomial influenza infections in cancer patients. Am J Infect Control.

[17] Poland GA, Tosh P, Jacobson RM (2005). Requiring influenza vaccination for health care workers: seven truths we must accept. Vaccine.

[18] Van Gageldonk-Lafeber AB, Dijkstra F, Van’t Veen H et al (2014) Lage influenzavaccinatiegraad onder ziekenhuismedewerkers. Ned Tijdschr Geneeskd 158:A7650 25308221

[19] European Centre for Disease Prevention and Control (2018) Seasonal influenza vaccination and antiviral use in EU/EEA member states – overview of vaccine recommendations for 2017–2018 and vaccination coverage rates for 2015–2016 and 2016–2017 influenza seasons. 10.2900/721517

[20] Lytras T, Kopsachilis F, Mouratidou E, Papamichail D, Bonovas S (2016). Interventions to increase seasonal influenza vaccine coverage in healthcare workers: a systematic review and meta-regression analysis. Hum Vaccin Immunother.

[21] To KW, Lai A, Lee KCK (2016). Increasing the coverage of influenza vaccination in healthcare workers: review of challenges and solutions. J Hosp Infect.

[22] Tsang TK, Lau LLH, Cauchemez S, Cowling BJ (2016). Household transmission of influenza virus. Trends Microbiol.

[23] Royal Australian College of General Practitioners (RACGP) (2020) Physical distancing and good hand hygiene: Australian flu cases drop by more than 99%. In: 2020-06-05. https://www1.racgp.org.au/newsgp/clinical/physical-distancing-and-good-hand-hygiene-australi. Accessed 11 Jun 2020

[24] Bitsori M, Galanakis E (2015). Vaccine-preventable infection morbidity of patients with chronic kidney disease and cocoon vaccination strategies. Expert Rev Vaccines.

[25] Rowe SL, Tay EL, Franklin LJ, Stephens N, Ware RS, Kaczmarek MC, Lester RA, Lambert SB (2018). Effectiveness of parental cocooning as a vaccination strategy to prevent pertussis infection in infants: a case-control study. Vaccine.

[26] Swamy GK, Wheeler SM (2014). Neonatal pertussis, cocooning and maternal immunization. Expert Rev Vaccines.

[27] Wang B, Russell ML, Moss L, Fonseca K, Earn DJD, Aoki F, Horsman G, Caeseele PV, Chokani K, Vooght M, Babiuk L, Webby R, Walter SD, Loeb M (2016). Effect of influenza vaccination of children on infection rate in hutterite communities: follow-up study of a randomized trial. PLoS One.

[28] Vrieze H, Van Haaren K, De Wit R (2018) NHG / SNPG-Praktijkhandleiding Griepvaccinatie. https://www.nhg.org/sites/default/files/content/nhg_org/uploads/nhg_snpg-praktijkhandleiding_griepvaccinatie_2018_web.pdf

[29] Wumkes ML, van der Velden AMT, van der Velden AWG (2013). Influenza vaccination coverage in patients treated with chemotherapy: current clinical practice. Neth J Med.

[30] Memoli MJ, Athota R, Reed S, Czajkowski L, Bristol T, Proudfoot K, Hagey R, Voell J, Fiorentino C, Ademposi A, Shoham S, Taubenberger JK (2014). The natural history of influenza infection in the severely immunocompromised vs nonimmunocompromised hosts. Clin Infect Dis.

[31] Price SA, Podczervinski S, MacLeod K, Helbert L, Pergam SA (2019). Understanding influenza vaccination rates and reasons for refusal in caregivers and household contacts of cancer patients. Am J Infect Control.

[32] Boey L, Bosmans E, Ferreira LB, Heyvaert N, Nelen M, Smans L, Tuerlinckx H, Roelants M, Claes K, Derdelinckx I, Janssens W, Mathieu C, van Cleemput J, Vos R, Vandermeulen C (2020) Vaccination coverage of recommended vaccines and determinants of vaccination in at-risk groups. Hum Vaccin Immunother 00:1–8. 10.1080/21645515.2020.1763739 10.1080/21645515.2020.1763739 PMC7553698 32614656

[33] Lester RT, McGeer A, Tomlinson G, Detsky AS (2003) Use of, effectiveness of, and attitudes regarding influenza vaccine among house staff. Infect Control Hosp Epidemiol 24(11):839–844. 10.1086/502146 10.1086/502146 14649772

[34] Connolly T, Reb J (2012). Toward interactive, Internet-based decision aid for vaccination decisions: better information alone is not enough. Vaccine.

[35] Czajka H, Czajka S, Biłas P, Pałka P, Jędrusik S, Czapkiewicz A (2020). Who or what influences the individuals’ decision-making process regarding vaccinations?. Int J Environ Res Public Health.

[36] Yeung MPS, Lam FLY, Coker R (2016). Factors associated with the uptake of seasonal influenza vaccination in adults: a systematic review. J Public Health (United Kingdom).

[37] Harrison N, Poeppl W, Miksch M, Machold K, Kiener H, Aletaha D, Smolen JS, Forstner C, Burgmann H, Lagler H (2018). Predictors for influenza vaccine acceptance among patients with inflammatory rheumatic diseases. Vaccine.

[38] Glavier M, Puyade M, Roblot F, Rammaert B (2019). Vaccination of cancer patients treated with chemotherapy: a survey among general practitioners. Med Mal Infect.

[39] RIVM (2017) Annual report surveillance of influenza and other respiratory infections in the Netherlands: winter 2016/2017. 10.21945/RIVM-2017-0096

[40] RIVM (2018) Annual report surveillance of influenza and other respiratory infections in the Netherlands: winter 2017/2018. 10.21945/RIVM-2018-0049

