# Supplementary material for: Cocoon vaccination for influenza in patients with a solid tumor: a retrospective study
Source: Support Care Cancer. 2020 Nov 12;29(7):3657–66. doi: 10.1007/s00520-020-05883-2 (PMC7657941; doi:10.1007/s00520-020-05883-2)
Supplement: Supplementary file 1 — (DOCX 22 kb). [file 520_2020_5883_MOESM1_ESM.docx]

**Appendix 1: Questionnaire influenza vaccination**

Many patients have to be hospitalized because of flu symptoms (influenza infection). These patients are mainly vulnerable patients. This research will study whether vaccinating the household contacts of vulnerable patients can protect patients from being hospitalized with flu. We, therefore, ask you to fill in the following questionnaire. The first part consists of 16 questions about you as a patient and your vaccination status. The second part is about your household contacts and consists of 10 questions about each household contact.

If you do not understand a particular question, you can always ask the nursing staff for help.

Name:

Date of Birth:

1. What is your sex?

- Male
- Female

2. What is your age?

…..… years old

3. What is your living situation?

- I live independently (with or without household contacts)
- I have informal care
- I have home care
- I live in a nursing home
- Other:

4. With how many people do you live in the same house?

- I live alone
- With 1 person
- With 2 people
- With 3 people
- With 4 people
- With 5 people
- With more than 5 people: … people
- I live in a nursing home

5. Do you leave your house regularly (to do grocery shopping, to go to work, to visit friends or family)?

- Yes
- No, I do not leave my house
- Other:

Vaccination against the flu (influenza)

6. Did your general practitioner advise you to get vaccinated against the flu last winter (2018-2019)?

- Yes
- No
- I don’t know

7. Did you get vaccinated against the flu last winter (2018-2019)?

- Yes
- No (go to question 10)
- I don’t know

8. If so, who advised you to get the vaccination? You can give multiple answers.

- The general practitioner
- The oncologist
- A different specialist
- The media
- Own initiative
- Other:

9. Why did you choose to get vaccinated last winter (2018-2019)? You can give multiple answers.

- Because of my own health
- Because I was advised to do so
- I don’t know
- Other:

10. If you chose not to get vaccinated, what was the reason for not getting vaccinated? You can give multiple answers.

- I did not receive an invitation from my general practitioner
- I do not find it necessary
- I am principally against vaccinations
- I am afraid of the side effects
- I forgot about the vaccination
- Other:

11. Were you hospitalized when you received the flu vaccination?

- Yes
- No
- I don’t know

12. Have you ever been hospitalized with the flu?

- Yes
- No
- I don’t know

13. If so, was your flu diagnosed by a test?

- Yes
- No
- I don’t know

14. Do you take special measures to prevent catching the flu during the flu season, such as washing your hands more often or having less physical contact when greeting someone?

- Yes, always
- Yes, if I have visitors
- Yes, but only in the hospital
- Yes, if:
- No

15. If you answered yes, which measures do you take?

……………………………………………………………………………………………………………………

……………………………………………………………………………………………………………………

16. Have you been vaccinated against the flu in the past five years?

- Yes, once
- Yes, every year
- No
- I don’t know
- Other:

Please fill in a separate additional questionnaire for every household contact you live with.

**Household contact 1**:

1. What sex is your household contact?

- Male
- Female

2. What age is your household contact?

- 0-10 years old
- 11-20 years old
- 21-30 years old
- 31-40 years old
- 41-50 years old
- 51-60 years old
- 61-70 years old
- 71-80 years old
- 81-90 years old
- 91-100 years old

3. What is your relationship with your household contact?

- First degree family member (child, parent)
- Second degree family member (brother, sister, grandparent, grandchild)
- Third degree family member (great grandparent, great grandchild, nephew, cousin, uncle, aunt)
- Partner or friend
- Other:

Vaccination against the flu (influenza)

4. Was your household contact advised by a general practitioner to get vaccinated against the flu last winter (2018-2019)?

- Yes
- No
- I don’t know

5. Did your household contact get vaccinated against the flu last winter (2018-2019)?

- Yes (go to question 7)
- No
- I don’t know

6. If not, what was his/her reason not to get vaccinated? You can give multiple answers.

- He/she did not receive an invitation from the general practitioner
- He/she did not think it was necessary
- He/she is principally against vaccinations
- He/she is afraid of the side effects
- He/she forgot about the vaccination

7. If your household contact did get vaccinated, why did he/she choose to do so? You can give multiple answers.

- Because of my health
- Because of his/her own health (go to question 9)
- Other:

8. If your household contact got vaccinated because of your health, who advised your household contact to do so? You can give multiple answers.

- The general practitioner
- The oncologist
- A different specialist
- The media
- Own initiative
- Other:

9. If your household contact got vaccinated because of his/her own health, who advised your household contact to do so? You can give multiple answers.

- The general practitioner
- The oncologist
- A different specialist
- The media
- Own initiative
- Other:

10. Has your household contact been vaccinated against the flu in the past five years?

- Yes, once
- Yes, every year
- No
- I don’t know
- Other:

**Cocoon vaccination for influenza in patients with a solid tumor: a retrospective study**

M.J. Rensink^a^, H.W.M. van Laarhoven^b^, F. Holleman^a^

^a^*Department of Internal Medicine and* ^b^ *Department of Medical Oncology, Cancer Center Amsterdam, Amsterdam University Medical Centers (UMC), University of Amsterdam, Meibergdreef 9, 1105 AZ Amsterdam, The Netherlands*

Corresponding author:

M.J. Rensink, BSc

Department of Internal Medicine, Amsterdam University Medical Centers (AUMC), University of Amsterdam

Meibergdreef 9, 1105 AZ, Amsterdam, The Netherlands

e-mail: [m.j.rensink@amsterdamumc.nl](mailto:m.j.rensink@amsterdamumc.nl)

Journal: Supportive Care in Cancer
